# Personnel's Experiences of Phlebotomy Practices after Participating in an Educational Intervention Programme

**DOI:** 10.1155/2014/538704

**Published:** 2014-10-30

**Authors:** Karin Bölenius, Christine Brulin, Ulla H. Graneheim

**Affiliations:** Department of Nursing, Umeå University, 901 87 Umeå, Sweden

## Abstract

*Background*. Blood specimen collection is a common procedure in health care, and the results from specimen analysis have essential influence on clinical decisions. Errors in phlebotomy may lead to repeated sampling and delay in diagnosis and may jeopardise patient safety. This study aimed to describe the experiences of, and reflections on, phlebotomy practices of phlebotomy personnel working in primary health care after participating in an educational intervention programme (EIP). *Methods*. Thirty phlebotomists from ten primary health care centres participated. Their experiences were investigated through face-to-face interviews. Findings were analysed using qualitative content analysis. *Results*. The participants perceived the EIP as having opened up opportunities to reflect on safety. The EIP had made them aware of risks in relation to identification procedures, distractions from the environment, lack of knowledge, and transfer of information. The EIP also resulted in improvements in clinical practice, such as a standardised way of working and increased accuracy. Some said that the training had reassured them to continue working as usual, while others continued as usual regardless of incorrect procedure. *Conclusions*. The findings show that EIP can stimulate reflections on phlebotomy practices in larger study groups. Increased knowledge of phlebotomy practices improves the opportunities to revise and maximise the quality and content of future EIPs. Educators and safety managers should reflect on and pay particular attention to the identification procedure, distractions from the environment, and transfer of information, when developing and implementing EIPs. The focus of phlebotomy training should not solely be on improving adherence to practice guidelines.

## 1. Introduction

Collection of blood by venipuncture is one of the most frequent procedures in health care [1]. Results from specimen analysis are essential for diagnosis and treatment and have essential influence on clinical decisions. Errors in phlebotomy can lead to patient suffering and jeopardise patient safety [2, 3]. The present study interviewed phlebotomy personnel working in primary health care centres (PHCs) in Sweden. The focus was on their experiences of performing venipuncture after participating in an educational intervention programme (EIP).

Blood specimen collection is performed following a clinical decision and request for patient testing. Phlebotomy includes processes of patient identification and specimen collection, handling, transportation, and analysis, with the results eventually being reported back to the patient [4]. Phlebotomy is, in line with other practical skills in health care, a complex procedure. Theoretical knowledge and manual skills, accuracy, and caring comportment, as well as good interaction between the health care personnel and the patient are essential when performing complex procedures [5]. To increase patient safety, as well as give the patient the optimal attention, these skills should be performed with good ethical intentions, based on solid practical and theoretical nursing skills [5, 6].

Errors in laboratory medicine can occur in all the steps during the total testing process, but most errors (46–68%) occur during the preanalytical phase [3, 4, 7]. Previous studies have shown that blood specimen collection from the wrong patient, insufficient volume, and clotted specimens are common, and these errors may be a reason for rejection of specimens [3, 8]. Adherence to blood collection practice guidelines has been investigated [9–12] in Sweden using a validated questionnaire [9, 13]. These studies document several important preanalytical errors such as incorrect patient identification, incorrect test request management, and incorrect tube labelling. Therefore, these areas need to be given attention to improve patient safety [9–12].

One way of improving patient safety is by developing effective training programmes for health care personnel. Curriculum designers and instructors need to employ appropriate pedagogical strategies for these programmes [14]. Some educational programmes aiming to evaluate phlebotomy training focus on single steps in the blood specimen collection procedure [15, 16], such as impacts of causing stasis [16, 17] or mixing test tubes [15], based on the argument that focusing on one specific problem, such as avoidance of haemolysis, may be more effective, compared with addressing a range of procedural problems, in attempting to improve the phlebotomist's skills [18, 19]. However, most of the studies on EIPs on phlebotomy only include a small number of participants. During recent years the use of e-learning has increased, especially in rural areas, and it appears to be achieving positive outcomes [20]. Few studies have evaluated whether phlebotomy training courses in larger study groups increase adherence to guidelines and improve practices [21].

Based on several studies showing poor phlebotomy practices [9–12, 22], we developed and implemented an EIP to improve, update and sustain phlebotomy practices. Given restricted premises, the EIP focused on the implementation of phlebotomy guidelines according to the Swedish* Handbook of Health Care* [23] and how to avoid haemolysis as well [24]. The EIP consisted of three parts: (1) compulsory, pre-EIP studies of the national phlebotomy guidelines; (2) compulsory attendance at two lectures; and (3) six written examination questions (randomly chosen from 24 questions) on phlebotomy.

On evaluation of the EIP, we found minor to medium improvements in sample quality and phlebotomy guideline adherence [19, 25]. It is hoped that this qualitative study will add further depth to understanding of phlebotomy in general, and the EIP's effect on participants' phlebotomy practice in particular. To our knowledge, no study has hitherto described phlebotomists' experience of blood sample collection. Therefore, the aim of this study was to describe primary health care personnel's experiences of phlebotomy practices after participating in an EIP.

## 2. Method

We performed a qualitative, descriptive study based on face-to-face interviews analysed using qualitative content analysis [26].

### 2.1. Participants

This study includes phlebotomy personnel working in public health centres (PHCs) with differing working environments in the county council of Västerbotten in northern Sweden. Some of the phlebotomy personnel work only in PHCs' laboratory units and some work for PHCs doing home visits; others alternate between the laboratory unit and home visits. The sample included phlebotomy personnel who had completed a phlebotomy questionnaire in 2007, participated in an EIP in 2009-2010, and answered the same questionnaire as follow-up between September 2010 and June 2011. In total, 30 phlebotomy personnel from ten PHCs agreed to participate. They worked at different PHCs in urban or rural areas and varied in respect of gender (three were men), age, working years, and profession. The median age was 57 years (range 32–65 years), and median of PHC working years was 20 (range 1–37 years). Among the 30 participants, 18 were enrolled nurses, eleven were registered nurses, and one was a biomedical technician.

### 2.2. Interviews

The invited personnel were informed about the study by a postal letter, followed by a phone call asking them to participate. Individual interviews were performed by the first author (Karin Bölenius) at the participants' workplace during working time, 1-2 months after they had completed the follow-up questionnaire. Before the interview started, participants were informed about the aim of the study. The interview questions were open-ended, with reflective elements, and informal in nature. The interview guide addressed experiences of phlebotomy after participating in an EIP. The initial question was, “Could you please tell me about your experiences of phlebotomy after participating in the EIP?” The initial question was followed by open-ended questions about experiences of preparation procedures prior to specimen collection, patient identification procedures, handling of tubes, information search procedures, experiences of patient safety, and error reporting. The participants' descriptions and reflections were clarified by follow-up questions, such as “Tell me more about it” and “Please could you give me an example of that?” Finally, the participants had the opportunity to raise issues concerning phlebotomy or the EIP. Each interview lasted 17–44 minutes (median = 22 minutes). The interviews were tape-recorded and transcribed verbatim.

### 2.3. Data Analysis

The text was analysed using qualitative content analysis [26] with an inductive approach [27]. Content analysis is a method of analysing written or verbal communication in a systematic way [28] focusing on differences between and similarities within parts of the text and resulting in categories and/or themes [26].

The analysis was performed in several steps. Firstly, the interviews were read through to gain a sense of the whole. In this step, the tape recordings were also listened to in order to validate the text. Text that was not relevant to the aim of the study, that is, reflections on other forms of specimen collection, such as capillary or bacterial specimen collection, was excluded. Secondly, the text was divided into meaning units, that is, words, sentences, and paragraphs related to each other by content. Thirdly, the meaning units were condensed while still preserving their core and labelled with codes. The codes were compared for differences and similarities and sorted into eight categories at a manifest and descriptive level [29]. The codes were* identification procedure*,* distractions from the environment*,* lack of knowledge*,* transfer of information*,* a standardised way of working*,* accuracy in clinical practice*,* continuing as usual in the right way*, and* continuing as usual regardless of incorrect procedure*. In the next step, the categories were abstracted and formulated as three subthemes:* becoming aware of risks*,* achieving improvements in clinical practice*, and* feeling reassured to continue working as usual*.

Themes are threads of latent meaning running through several categories. After several discussions among the research team, consensus was reached and a theme was formulated:* education opens up opportunities for reflection on safety*. In addition to discussing the codes, categories, and theme, we present below a number of relevant quotations along with our findings to allow the reader to judge the authenticity of our interpretations.

### 2.4. Ethical Considerations

The research plan was approved by the Regional Ethical Review Board (Dnr 2010-355-32M, additions to Dnr 06-104M). All participants received verbal and written information on the study. Participants gave their informed consent to participate and were able to choose the time and place of the interview. Also they had the opportunity to stop the interview if they wished. Furthermore, the participants were reassured that all information would be handled confidentially and that participants' identity would not be revealed in the final results.

## 3. Results

### 3.1. Education Opens Up Opportunities for Reflection on Safety

In this study we found that* Education opens up opportunities for reflection on safety*. This means that the EIP made participants aware of risks of and led to improvements in phlebotomy practice, and further reassured them regarding the phlebotomy procedure (Table 1).

### 3.2. Becoming Aware of Risks

The participants reported that the EIP had made them aware of risks in relation to the* identification procedure, distractions from the environment, lack of knowledge, and transfer of information*.

#### 3.2.1. The Identification Procedure

Participants described situations in which they sometimes left a patient alone before the blood collection was finished. This meant that they might forget to label the tubes or ask for the patient's identification number on returning to the room. They also reflected that failure to follow identification procedures can lead to inaccuracy. Verifying the identity of someone they knew might feel unnecessary and might sometimes even be awkward or embarrassing; however, it needed to be done. Other experiences relating to the identification procedure were that identification of patients was affected by communication problems. The participants gave the example of difficulties in identifying immigrants, children, and people suffering from dementia. Experiences of risky identification practices are cited below:

*It happens, an incident now and then, that one puts a label and never asks, one never asks [about the patient's name and identification number]. It's important, because I can paste the wrong label. You cannot assume that this is the correct label without checking with the patient—‘Is this you?' (Interview 27)*

#### 3.2.2. Distractions from the Environment

The participants described distractions from the surroundings. The phlebotomist's physical work environment varied, from a phlebotomy room to completely unfamiliar places. Rooms allowing blood specimen collection from several patients simultaneously were described as presenting a risk for errors and also as jeopardising patient integrity. Sampling at a patient's home increased the risk of forgetting collection materials. Late orders and working under time pressure in a stressful and noisy atmosphere were common and also put the patients' safety at risk. The participants related that they were sometimes asked to register and sign test request forms from the municipality, when they had no control over the collection quality. Thus, they deviated from phlebotomy guidelines by signing for others, which felt wrong. On the other hand, not taking responsibility for their coworkers' work could lead to suffering for the patient.

Two distractions from the environment are described below:

*Everyone will pass by [the lab], although they might not come to have samples taken … Doctors ask a lot of different things. It need not be about sampling, but it could be other things they want to know, about patients, reservations and appointments … and there are people who call and are looking for doctors …. It can affect patient safety in some cases. Because it is really stressful, so, when it becomes crazy, then I will be honest and say. (Interview 20)*

It sometimes happened that parents who were in a hurry became angry because they had to wait for the analgesic to take effect on their child prior to sampling. There were also participants who sometimes had to hold a patient still during phlebotomy, which caused conflicting emotions for the participants:

*Yes, it is …. It feels almost like abuse against the person that you are caring for, but at the same time, it's a safety issue for me, because I only need to make a puncture once if a staff member is holding the patient's hand steady. You can do the collection directly, rather than having to try yourself, and the patient moves, and you must puncture them several times …. (Interview 14)*

#### 3.2.3. Lack of Knowledge

The participants reflected on lack of knowledge among phlebotomy personnel and described this as putting patients at risk. They also reported that sometimes, prior to the EIP, they had kept the phlebotomy tubes in their handbags, unaware of possible consequences. Also, how to label tubes and perform phlebotomy with a number of tubes using the correct order of draw had been new for several of the participants. After the EIP, the participants understood that test tube additives can be transferred between tubes and that shorter tourniquet use gives more reliable test results and less suffering for patients. Not knowing the guidelines and rarely performing phlebotomy were described as risky. Sometimes participants had to recall patients for repeated sampling. This felt bad and was often due to someone else's mistake.

An example of lack of knowledge is given below:

*Nurses have some problems in the summer when we do not perform sampling. The phone rings every five minutes and they ask about a particular analysis and what it means. What is it? And like that …. The patient should be fasting, I reply. ‘Fasting', she says. ‘I am in the patient's home and she has eaten.' Yes, it is fasting and we should just know. You need the knowledge when you are at home with the patient. (Interview 29)*

#### 3.2.4. Transfer of Information

The participants pointed out that the transfer of information presented a risk for misunderstanding. Sometimes patients and/or the professionals received wrong information or no information at all, which might impact patient safety. Sometimes, when a patient came to the PHC for sampling, no order was available for that patient. In other cases, information and a referral had reached the patient but not the PHC staff. It could happen that the wrong test was ordered or entered onto computer. Often the phlebotomist had no control over this, since it was a physician, another colleague, or staff from another ward who often initiated the order.

*It is not difficult to make the sample collection or deal with the patients, but other things can be difficult. It can take a very long time when it is not mentioned if the patients have referrals from other clinics and there is no information about which analysis has been ordered, or it is a weird order that nobody knows about …. And to call and hunt down personnel at the clinic, or even to call the lab and find the person who performs the analysis … (Interview 20)*

### 3.3. Achieving Improvements in Clinical Practice

Participants reflected on safety and described how they had achieved improvements in clinical practice in relation to a* standardised way of working* and* accuracy in clinical practice*.

#### 3.3.1. A Standardised Way of Working

To ensure quality, a standardised way of working was described as important, particularly in stressful or emergency situations. After having undergone the training, the participants described improvements in using the practice guidelines. As one said,

*[I've changed] the order of the test tubes, I believe. That is the first thing I think of that has changed since the training. And to avoid stasis if possible. (Interview 2).*

Participants also reported that since the EIP they had used the tourniquet for shorter periods, and they had changed details like the order of draw, in line with the national guidelines. They said they had had no problem changing procedure as the new methods were easy to understand and practical. They also reported that they had improved the preparation procedures for the PHC together with coworkers. One PHC had bought bags especially for phlebotomy, with space for all materials and a carrier to store the tubes standing as instructed. The participants described better routines, such as performing one thing at a time. If the phone rang, it was better to answer after finishing the sampling. This improved patient safety. The participants also reflected on developments in planning—being well prepared, making systematic checks to ensure that you have all the material available, and not having to fetch anything during sampling. Since the EIP, participants had used the internal network more frequently when searching for specimen collection instructions; and they found that they had to ask colleagues less often. In addition to increasing patient safety, they also remembered to take all phlebotomy materials with them to the home of a patient. Patients who were sampled frequently were allowed to take care of their own referral labels, such as the name and birth registration number. A standardised way of working is outlined below:

*I always take standardised samples … I prepare what I can and I tell the patients … and so we go and take samples. The referrals are already prepared …. Then it's simple … I label the test tubes and referrals and so on …. You should have it done in advance, in fact, and yet, if it is children, it takes time. There may be stressful situations, and then it is particularly careful to work according to procedures. (Interview 28)*

#### 3.3.2. Accuracy in Clinical Practice

A standardised way of working contributed to increased accuracy in clinical practice. Increased accuracy ensured patient safety, gave more accurate test results, and meant that repeated blood specimen collection was required less often. Participants reported that since the EIP, they had become more careful as they had learned about the importance of verifying the patient's identity against the test request. They recalled that previously they had approached the identification procedure fairly casually. The EIP motivated them to adhere to guidelines and had reminded them of the consequences of being careless. One nurse said that her efforts to increase accuracy during the patient identification procedure were the result of others' changed behaviour in this regard and not of her own increased awareness. Improved accuracy in most cases meant labelling tubes in the presence of the patient and in accordance with the guidelines. The participants also described being more careful in general:

*I'm a little more careful to always finish everything when the patient is in here. That I have changed. Before, I used to let the patient walk out of the room without being ready. Today, I tell them to stay until I'm done. All referrals are completed, everything is ready, and I have posted the date and removed the name from the computer … and I always bring just one patient at a time into the room … (Interview 16)*

### 3.4. Feeling Reassured to Continue Working as Usual

Feeling reassured to continue working as usual included* continuing as usual in the right way *and* continuing as usual regardless of incorrect procedure*. Some participants felt that they were already working as instructed, while others thought that they had not learnt anything new during the EIP or just did not want to change their routines.

#### 3.4.1. Continuing as Usual in the Right Way

Participants in this group felt reassured that they were already working as instructed. Some said that the EIP had not taught them anything new and that patient identification procedures had always been important to them. Some reported that they had achieved updated knowledge and that the EIP had motivated them and reminded them of possible risks and complications in phlebotomy. With regard to ethical issues, they said it was no problem to adapt work according to different situations and find new information from the internal network. Phlebotomy had been made visible by the EIP. They thought that all phlebotomy personnel should receive the phlebotomy training, regardless of background, to ensure patient safety. After many years without training, the participants appreciated having been able to participate in the EIP:

*For me, it feels really good …. In the 70s there was no education. The more education, the more I understand about what could go wrong …. Yes, it is very positive … Yeah, it's like my job is also important. (Interview 16)*

#### 3.4.2. Continuing as Usual Regardless of Incorrect Procedure

On the other hand, some participants did not change their routines; they continued as usual despite the knowledge that they were not following correct procedure. After the EIP, these participants reported that they were still not working according to the new instructions. For example, one participant continued working without gloves, not thinking about safety. Instead, this phlebotomist thought of patient comfort and, also, of avoiding repeated sampling through accuracy in finding veins ungloved. Other participants said they had no intention of using the web-based internal network, as they would soon retire. The participants described changes in referral and identification procedures due to general development. The quotation below is a response to the question, “Have you changed anything since the EIP?”

*No, I didn't get so much out of it … I use gloves as little now [as before the education]. Gloves are for me and my safety, but I am here for the patient's sake. (Interview 29)*

## 4. Discussion

Education opened up opportunities for reflection about safety. This was the main finding of our study. More specifically, participants in this study became aware of risks, experienced improvements in clinical practice, and felt reassured about the work they were doing.

The objective of the EIP in this study was to improve phlebotomy personnel's adherence to practice guidelines with the aim to decrease errors. In addition, from a system and a patient perspective, all personnel involved should take responsibility for mistakes in health care [30, 31], resulting in improved overall care and ensuring patient safety. Phlebotomy is performed similarly across the whole Swedish PHC system and is regulated by the Swedish National Board of Health and Welfare [32] and national guidelines [23], which indicates that there is probably the same risk for errors in other Swedish county councils. Our results suggest that educators, and safety managers, should focus on the identification procedure, distractions from the environment, and transfer of information, when developing and implementing EIPs, and should not focus solely on improving adherence to practice guidelines.

*Transfer of information* between, for example, the county council and the municipality was described as a risk in our study. Transfer errors can be explained by deficiencies in the organisational structure [33, 34], for example, communication failures [31], but this has not been addressed in our present study. In a previous intervention study performed by our research group [25], phlebotomists from the intervention group reported less use of printed, presumably outdated instructions and more use of information via the internal network. However, for implementation of an intervention aiming to improve transfer of information between units or people, more research is needed.

Primary health care centres should offer a work environment to ensure patient and personnel safety [30]. Participants identified* distractions from the environment* as a risk for phlebotomy errors that might jeopardise patient safety. For instance, a phlebotomy room containing three patient chairs was described as a source of stress for phlebotomy personnel, with consequences for patient integrity. To work in a calm and silent physical environment is important [35], and to perform practical skills with fluency and without interruptions is crucial for good performance [5, 36]. The work environment can be seen as a resource that makes actions possible, but it can also be a source of stress [35].

Some of our participants did not change their behaviour. Their inaction may be explained by low motivation to change. For example, one participant argued that gloves were only important for the phlebotomist's own safety, not for patients' safety. When personnel are supported by the local work management they may have better opportunities to improve their skills, and motivation is an important factor in influencing personnel behaviour [37].

Following the EIP, one PHC changed their routines at a local level by giving those patients who frequently had to have phlebotomy the responsibility for their own referral labels, including their name, identity number, and address. It was hoped that this would decrease occurrences of incorrect names on the paper referrals, for example. The participants and the work management were therefore attentive to the needs of the patient as a whole, when integrating them into the care [5, 36].

Errors performed by frontline personnel could eventually be avoided by improved knowledge and awareness [30]. Our findings show that the EIP made participants aware of risks related to* patient identification* and* lack of knowledge* and thus taught them to better adapt their practices to the individual person or situation. De Leval and coauthors [38] report that personnel with high awareness of safety may also be more successful in dealing with eventful circumstances. Participants in this study achieved improvements in clinical practice; they pointed out that they were more diligent in accurately identifying a patient. These results are confirmed by our previous study showing that phlebotomy personnel significantly improved control of patients' photo identification [25]. Unsafe practice during patient identification procedures could lead to blood specimen collection taken from the wrong patient [8]; therefore, the identification procedures are of importance to ensure patient safety. Patient identification has to be performed correctly and exactly and in accordance with regulations [32]. Furthermore, accuracy is important in all steps of a practical skill [5, 36].

The participants also improved by adopting a* standardised way of working*. For example, they described shorter application of the tourniquet, which is in line with our previous intervention study [25]. Prolonged tourniquet application was also related to incorrect order of draw [5, 36]. Use of venous stasis before cleaning the patient's skin means that tourniquet application will probably be longer than 1 minute [39].

In this study, some ethical issues must be highlighted. Our results show that participants reflected on* distractions from the environment* and their effect on the phlebotomy procedure. They described conflicting emotions when they had to hold a patient still, deviate from instructions, or take responsibility for other people's work by signing for them or when parents tried to interfere with the sampling procedure in a child. This indicates that phlebotomists' ethical practice is a complex process of reasoning and decision making which is also influenced by personal and contextual factors [40]. Ethical decisions are often based on medical and nursing knowledge and on individual values and experiences [40, 41]. Our participants described* lack of knowledge* as a risk for errors; lack of knowledge may affect how phlebotomists act and take decisions in different situations. Ethical decisions are also influenced by collaboration with colleagues, as phlebotomists and nurses generally seek to conform to the views of other nursing personnel and often put their own opinions aside [40]. One participant related that she had become more accurate in identifying patients, not out of increased awareness but in order to adhere to other colleagues' opinions.

To enhance trustworthiness, all the interviews were conducted by the first author (Karin Bölenius) at the participants' workplace and using the same open-ended guide. A strength point of the study is that we conducted 30 interviews in ten PHCs, which provided a variety of experience of phlebotomy personnel. A limitation of the study may be that the interviews were performed in different places and could have been affected by the atmosphere of the workplace in a few cases [29]. However, the authors' view is that the participants felt free to express their experiences. Furthermore, the interviews were conducted 1-2 months after the follow-up questionnaire, which may have reduced the transferability of evaluating the educational programme but not their experiences of phlebotomy. Recall bias can influence interview answers.

To achieve dependability, all authors discussed every step of the analytical process and tested the stability of meaning units and categories until consensus was reached. We further reflected on our findings in relation to the interview text. Krippendorff argues that a text never implies one single meaning [28]. Therefore, this is one possible interpretation of health care personnel's experiences that education opens up opportunities for reflection on safety.

## 5. Conclusion

The results show that EIPs can stimulate reflection, in larger study groups, on phlebotomy practices. Increased knowledge of phlebotomy practices as well as participant feedback improves the opportunities to revise and maximise the quality of EIPs with relevant content. Participants in this study had become aware of risks and had achieved improvements in clinical practice as a result of the EIP and felt reassured by the training. Areas that were identified as needing more focus are the patient identification procedure, distractions from the environment, and transfer of information. This should be borne in mind when developing and implementing EIPs. In other words, the focus should not be solely on improving adherence to practice guidelines. Our suggestions for improving phlebotomy practices are, firstly, to improve the work environment so as to decrease disturbances and ensure patient integrity. Secondly, we suggest investigating and improving the transfer of information between different occupational groups and units. By thus supporting the personnel and taking their experiences into account we could motivate personnel discussions. Important issues arising in personnel discussions should be prioritised when developing and implementing phlebotomy EIPs in the future.

## Figures and Tables

**Table 1:** Overview of the categories, subthemes, and theme revealed during the analysis.

Categories	Subthemes	Theme
Identification procedure	Becoming aware of risks	Education opens up opportunities for reflection on safety
Distractions from the environment		
Lack of knowledge		
Transfer of information		
A standardised way of working	Achieving improvements in clinical practice	
Accuracy in clinical practice		
Continuing as usual in the right way	Feeling reassured to continue working as usual	
Continuing as usual regardless of incorrect procedure		
